# Associations Between Temporomandibular Pain and Biobehavioral Variables in Dental Students in Response to an External Stressor

**DOI:** 10.11607/ofph.3239

**Published:** 2023-11-17

**Authors:** István Somoskövi, Márta Radnai, Richard Ohrbach, Tímea Dergez, István Tiringer, Andrea Radácsi, Ákos Nagy

**Affiliations:** Department of Dentistry; University of Pécs Medical School; Department of Prosthodontics; Faculty of Dentistry; University of Szeged; Department of Oral Diagnostic Sciences; University at Buffalo; Institute of Bioanalysis; University of Pécs Medical School; Department of Behavioral Sciences; University of Pécs Medical School; Department of Dentistry; University of Pécs Medical School

**Keywords:** anxiety, depression, oral behaviors, psychologic stress, temporomandibular pain

## Abstract

**Aims:**

To assess changes in temporomandibular disorder (TMD) pain and multiple biobehavioral variables relevant to TMDs in response to an external stressor.

**Methods:**

Self-reported data using online DC/TMD questionnaires were collected from volunteer dentistry graduate students. Data collection was performed on two occasions: during a non-exam period of the semester and during the subsequent exam period. Changes in the proportion of students with pain, differences in pain grade, and severity of biobehavioral status were measured and compared over the two periods. The association between severity of non-exam–period biobehavioral status and pain presence was also tested to assess whether biobehavioral variables can predict pain occurrence or persistence. Chi-square test, Wilcoxon signed-rank test, ANOVA, and Kruskal-Wallis tests were used for data analysis. *P* < .05 was considered significant.

**Results:**

Of the 213 enrolled students, 102 remained after data reduction. In the non-exam period, the proportion of individuals with pain was 24.5%; in the exam period, the proportion was 54.9%, and more students had a higher pain grade. The severity of all biobehavioral variables was higher in the exam period, but there was no association between changes in the presence of pain and changes in biobehavioral variables. Higher anxiety and parafunction levels were found in those who reported pain on both occasions.

**Conclusion:**

Exam periods initiate readily measurable changes in the psychologic status of many students, as well as alterations in their temporomandibular pain. Higher levels of anxiety and oral behaviors during non-exam periods seem to be predictors for persisting pain. *J Oral Facial Pain Headache 2023;37:167–176. doi: 10.11607/ofph.3239*

Facial pain is the dominant and most distressing symptom of temporomandibular disorders (TMDs), while joint sounds and disturbed mandibular movements are the next most common.^1^ Pain can occur in the temporomandibular joint (TMJ), the masticatory muscles and surrounding tissues, or both, and referred pain can occur in additional locations on the face, head, and neck.^2^ Painful TMDs can become chronic, currently defined as pain for 3 months or longer.^3^ The reported prevalence estimates of painful TMDs when using consistent study designs suggest that approximately 5% to 12% of the population is affected across all studied countries,^4–6^ putting a significant burden on both patients and the health care system. Therefore, TMD pain has a huge impact on an individual’s quality of life,^7^ affecting them, their family and, ultimately, society.

Earlier beliefs regarding the etiology and pathomechanisms of TMDs focused on jaw-specific factors, but current evidence indicates that painful TMDs have many characteristics similar to other chronic pain conditions, such as low back pain and headaches. These similarities include psychologic factors such as anxiety, somatic symptoms, and depression,^8^ as well as general health-related factors such as poor sleep. While psychologic distress is suspected to play a role in the onset and progression of TMD pain, distress can also be a consequence of TMDs.^9,10^ Moreover, in evaluating the causal determinants of TMD, the OPPERA (Orofacial Pain: Prospective Evaluation and Risk Assessment) project revealed that numerous psychosocial variables can predict first-onset TMD pain.^11^

An important source of distress is psychologic stress,^12^ and university students are repeatedly exposed to increased levels of stressors^13,14^ such as study or work problems. For example, very high prevalence rates of depression, anxiety, and stress were reported among medical students when measured during the regular study period.^15^ During exam periods, student distress was considerably higher, with consequences sufficient to cause fundamental changes such as measurable alterations in cardiovascular parameters.^16^ Severe distress can manifest as depression, burnout, alcohol abuse, and suicidal thoughts, and it has a significant impact on medical students’ personal wellbeing.^17^ Consequently, recurring stressors—despite seeming to appear as adequately managed—are important risk factors for a range of functional disorders among younger adults.

The purpose of the present study was to evaluate the presence and severity of TMD pain as well as the severity of biobehavioral variables (anxiety, depression, jaw functional limitation, and oral parafunction) in response to an external stressor, ecologically implemented as an exam period contrasted against a non-exam period. Both pain and biobehavioral factors were evaluated using self-report questionnaires; clinical examination was not implemented. The following hypotheses were tested: *(1)* There is significant worsening in the measured variables from the non-exam to the exam periods; *(2)* The extent of anxiety, depression, and oral parafunctions at enrollment act as predictors for the onset of pain during the exam period; and *(3)* A direct association can be found between changes in the extent of biobehavioral variables with subsequent frequency and severity of pain.

## Materials and Methods

Participants were recruited from the students attending the Faculty of Dentistry, University of Pécs Medical School, Pécs, Hungary. Prospective participants were contacted via email and invited to volunteer in the observational study. Students in the first year of their studies were excluded, as such students are expected to have levels of psychologic distress more strongly related to starting university. The expected sample size was based on previously published studies with a similar sample size; for example, Resende et al^7^ enrolled 120 individuals with an effect size of 0.77 for anxiety levels in patients with and without TMDs. All participants were required to read and sign a consent form upon enrollment, and an information sheet explaining what would be measured and the timing of the study was given to each student. Data collection was anonymous; students were asked to provide a username of their choice, and because data collection was performed two times, respondents were asked to provide the same username on both occasions to link their data. The study protocol was approved by the Regional and Institutional Research Ethics Committee (ID: 6448-PTE2020).

Data collection was based on self-report using the Hungarian version of the DC/TMD questionnaires.^18^ The presence of orofacial pain over the prior 30 days was assessed using the TMD Pain Screener, which is specific for the evaluation of temporomandibular pain. This instrument is a brief three-item screener intended to be used in research as well as in clinical applications. A study conducted to assess validity of the Pain Screener found excellent reliability (α = 0.87), sensitivity (99%), and specificity (97%) values.^19^

Pain intensity and pain-related disability were assessed using the corresponding variables in the Graded Chronic Pain Scale (GCPS), version 2.0, adapted for assessing a 30-day reference time period.^20^ Psychologic variables of depression and anxiety were evaluated using, respectively, the Patient Health Questionnaire-9 (PHQ-9)^21^ and Generalized Anxiety Disorder-7 (GAD-7).^22^ The Jaw Functional Limitation Scale 8-item version (JFLS-8) was used to assess the overall functional status of the masticatory system.^23^ For the evaluation of oral parafunctional behaviors, the Oral Behaviors Checklist (OBC) was administered.^24^

Both Axis I and II questionnaires were administered online to the students on two separate occasions, once during the second semester of academic year 2019/2020 (instruction period, T1) and approximately 3 months later in the subsequent exam period (T2). The reference time frames in the questionnaires are 1 month (30 days, for example, TMD Pain Screener) or 2 weeks (for example, GAD-7). Consequently, the two administrations of the data collection instruments were set at approximately 32 days after the beginning of the semester for T1 and approximately 32 days after the beginning of the 7-week exam period for T2. In this way, a maximum instrument reference period of 30 days was inclusive of the beginning of the semester for the T1 data, and similarly the reference period of 30 days was inclusive of the first part of the exam period for the T2 data. 

For dentistry students, the T1 period is characterized by attending lectures and seminars and by taking part in clinical and preclinical (laboratory) practices. These events are timetable-based, so students follow a relatively constant daily routine, and mid-term tests or exams are infrequent. In contrast, in the exam period (T2), preparation and exam situations occur repeatedly over approximately one and a half months, and a disrupted time schedule is also distinctive. Because this sample included students from all academic years (except the first year), they were in different points in the exam period. The number of exams vary each exam period, and students are mostly free to organize their own examination schedule. Such exam periods are universally regarded as stressful for most individuals, and the exam period was used in this study as an ecologically valid stressor to test the study hypotheses.

### Data Reduction and Statistical Methods

Four groups were created based on the presence or absence of pain at T1 and T2. The presence of TMD pain in the last 30 days and whether pain was modified by jaw activity were determined using the TMD Pain Screener instrument. When the student reported a positive response to both questions, the student was classified as having TMD pain; a “no” response to either question resulted in a classification of not having TMD pain. Consequently, the four groups were labeled as follows: “no pain” = no pain at either T1 or T2; “new pain” = no pain at T1, pain at T2; “remitted pain” = pain at T1, no pain at T2; and “continuous pain” = pain at both T1 and T2.

Age, sex, and the proportion of individuals with pain were presented as simple descriptive statistics. Characteristic Pain Intensity (CPI), Interference Score (IS), and Disability Points (DP) were calculated from the GCPS. Based on pain intensity and pain interference as well as the number of disability days, students were assigned to a grade of pain as described by Von Korff et al^25^: Grade 0 = no pain (CPI = 0, DP N/A); Grade 1 = low intensity pain, without disability (CPI < 50, DP < 3); Grade 2 = high intensity pain, without disability (CPI ≥ 50, DP < 3); Grade 3 = moderately limiting (CPI N/A, DP = 3 to 4); and Grade 4 = severely limiting (CPI N/A, DP = 5 to 6).

Total sum scores were calculated for both depression and anxiety, and students were classified as normal (0 to 4 points), mild (5 to 9 points), moderate (10 to 14 points), moderately severe (15 to 19 points), and severe (20 to 27 points) for depression and as normal (0 to 4 points), mild (5 to 9 points), moderate (10 to 14 points), and severe (15 to 21 points) for anxiety, according to the interpretation guidelines.^21,22^ Similarly, for the JFLS, summary scores were computed and compared between T1 and T2. For the OBC, sleep-related and waking-state behaviors were scored separately as a weighted sum. JFLS and OBC scores were interpreted as noncategorical data. T1 anxiety, depression, and sleep-related and waking-state oral behavior scores were compared in the TMD pain groups to assess the possible predictive value of these measures taken before the active stressor period. Changes between T1 and T2 scores of the biobehavioral domain variables (ie, anxiety, depression, jaw functional limitation, oral behaviors) were compared in the different pain groups to assess whether the process of change provided further insights into who developed pain.

Categorical data were presented as number and percentage, while quantitative data were presented as mean, SD, median, and ranks. For categorical variables, chi-square test was used for comparisons between groups. Wilcoxon signed-rank test was used for comparisons between two related groups in case of data pairs of quantitative data and nonparametric distribution.

Kolmogorov-Smirnov test was used to analyze the distribution of variables. One-way analysis of variance (ANOVA) was used to test the difference among groups regarding changes in anxiety, depression, jaw functional limitation, and oral behavior scores from T1 to T2. These variables follow a normal distribution.

For T1 anxiety, depression, and oral parafunction, distribution of the residuals was not normal, and their associations with the changes in presence of pain from T1 to T2 were tested using independent-samples Kruskal-Wallis test.

All analyses were two-tailed, and *P* < .05 was considered significant. IBM SPSS software (version 26) was used.

## Results

**Fig 1:**
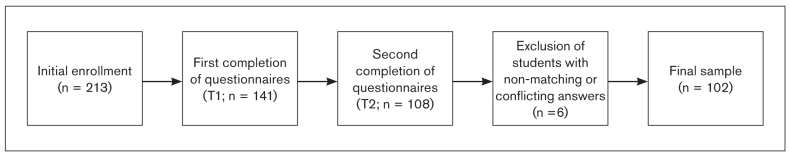
Flowchart showing the number of students who participated in the study from initial enrollment to final sample.

Initially, 213 undergraduate students were enrolled, and 141 students completed the questionnaires at T1. Of those who completed the questionnaires at T1, 33 did not respond at T2, and 5 students could not be matched at T2. The responses in the Pain Screener and GCPS were conflicting from one student who was removed from the analyses, leaving a final sample of 102 individuals who provided valid responses to all questionnaires on both occasions (Fig 1). The dropout rate from T1 to T2 was 28%. The mean age of the 102 students was 23.7 years (20 to 31 years) with a sex distribution of 62.5% female.

The variance in proportion of students regarding the presence of pain at T1 and T2 is shown in Fig 1.

**Fig 2:**
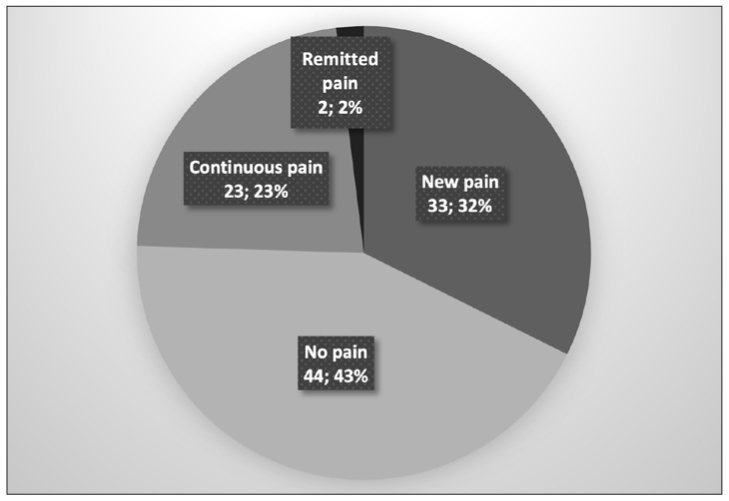
Pie chart showing the number and percentage of students in the TMD pain groups.

Twenty-five students (24.5%) reported pain at T1, and 23 of them also reported pain at T2. Of the 77 students who had no pain at T1, 33 (43.3%) developed pain by T2, and 44 students remained pain-free at T2 (Fig 2). Because the “remitted pain” group contained only 2 students, this group was dropped from further analyses based on the pain group comparisons.

Considering the full sample, CPI and IS scores were significantly higher at T2 than T1 (*P* < .001 for CPI and *P* = .003 for IS, Wilcoxon test). When analyzing the “continuous pain” group (who had CPI > 0 at both T1 and T2, by definition) alone, a trend toward higher CPI and IS scores at T2 was observed, but the difference was not significant (*P* = .098 for CPI and *P* = 1.000 for IS; Table 1).

**Table 1:** Changes in Characteristic Pain Intensity (CPI) and Interference Score (IS) in the No Pain, New Pain, and Continuous Pain Groups

	CPI			IS		
	No pain	New pain	Continuous pain	No pain	New pain	Continuous pain
T1	0	0	23.6 (12.1)	0	0	6.4 (9.2)
T2	0	23.5 (16.5)	31.1 (14.5)	0	8.4 (14.9)	6.8 (9.8)

Data are reported as mean (SD). Significantly higher CPI (*P* < .001) and IS (*P* = .003) scores were observed at T2 than T1 in the full sample.

**Fig 3:**
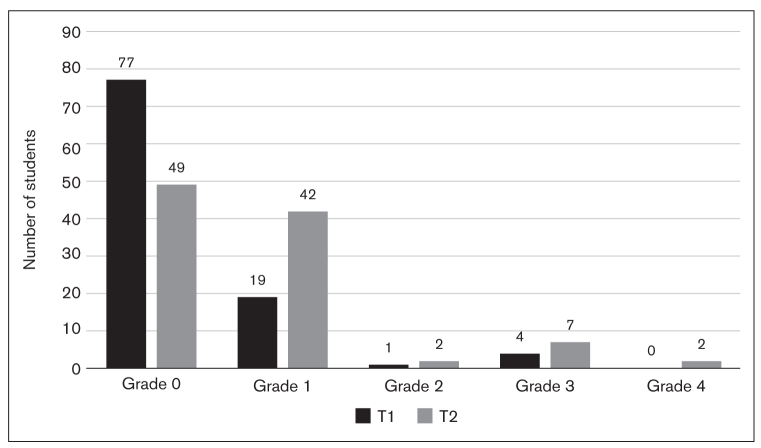
Bar graph showing students with different levels of pain during the non-exam period (T1) and the exam period (T2).

Based on the CPI and DP scores, it was found that while 75.5% of students had no pain (and therefore no pain-related interference; Grade 0) at T1, the proportion of these students decreased to 48.0% by the T2 assessment. Correspondingly, the proportion of those in Grade 1 increased considerably from 18.6% to 41.2%. At T1, only 4 students presented with moderate interference (Grade 3) and none with severe interference (Grade 4), whereas 9 students were categorized as Grade 3 or 4 at T2 (Fig 3).

**Fig 4:**
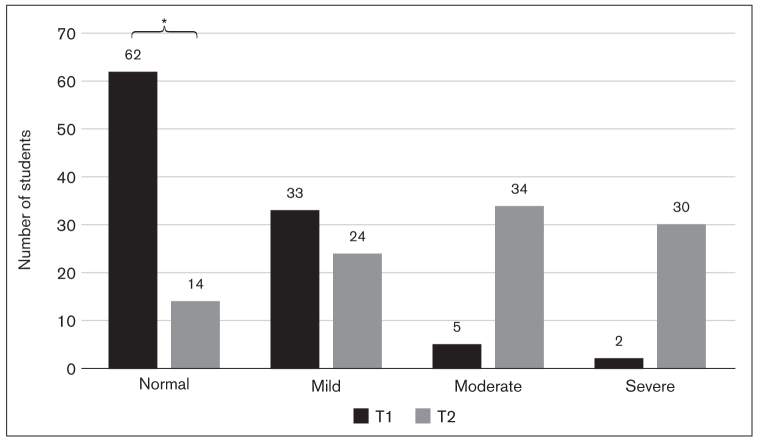
Bar graph showing students with different levels of anxiety during the non-exam period (T1) and the exam period (T2) based on total sum scores of GAD-7: normal = 0–4 points, mild = 5–9 points, moderate = 10–14 points, severe = 15–21 points. The number of students in the normal group was significantly lower at T2 than at T1 (*P* < .001).

**Fig 5:**
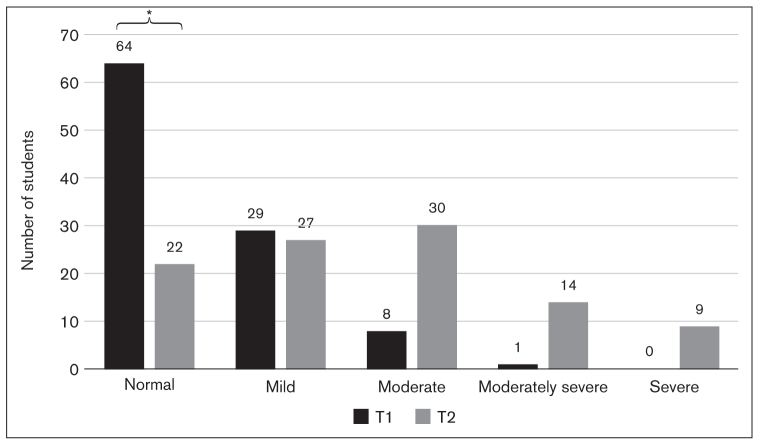
Bar graph showing number of students with different levels of depression during the non-exam period (T1) and the exam period (T2) based on total sum scores of the PHQ-9: normal = 0–4 points, mild = 5–9 points, moderate = 10–14 points, moderately severe = 15–19 points, severe = 20–27 points. The number of students in the normal group was significantly lower at T2 than at T1 (*P* < .001).

Significantly worse anxiety and depression levels were found at T2 compared to T1 (*P* < .001 for each of anxiety and depression, Wilcoxon test). In terms of anxiety, while 93.2% of students were classified as normal or mild at T1, their proportion had reduced significantly by T2 with the corresponding increase in moderate and severe anxiety classes (*P* < .001, chi-square test; Fig 4). Similarly, students with normal or mild depression represented 91.1% of the total sample at T1 but only 48.1% at T2; correspondingly, a significant increase in the number of students with moderate or worse depression was observed (*P* < .001, chi-square test; Fig 5). At the person level, these changes can be explained in two ways: Students in the normal group at T1 developed mild anxiety/depression by T2, while those in the mild group worsened to moderate or severe; or the distribution of the normal grade at T1 was spread across all three mild, moderate, and severe levels.

**Fig 6:**
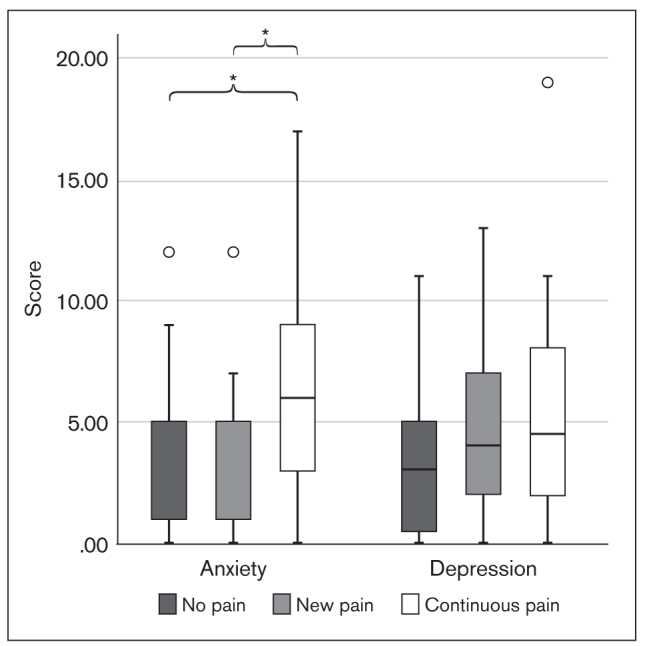
Box plot showing T1 anxiety and depression scores in the TMD pain groups (*P = .010 for anxiety and *P* = .143 for depression; independent-samples Kruskal-Wallis test).

Significant differences were found across the three groups of students regarding the extent of anxiety at T1 (*P* = .022, independent-samples Kruskal-Wallis test), and pairwise comparisons demonstrated that those in the “continuous pain” group presented a significantly higher level of anxiety symptoms than the other two groups, “new pain” and “no pain” (*P* = .010; Fig 6). No difference was found between the extent of depression at T1 and the presence of pain in any of the three groups (*P* =.143, independent-samples Kruskal-Wallis test; Fig 6).

**Fig 7:**
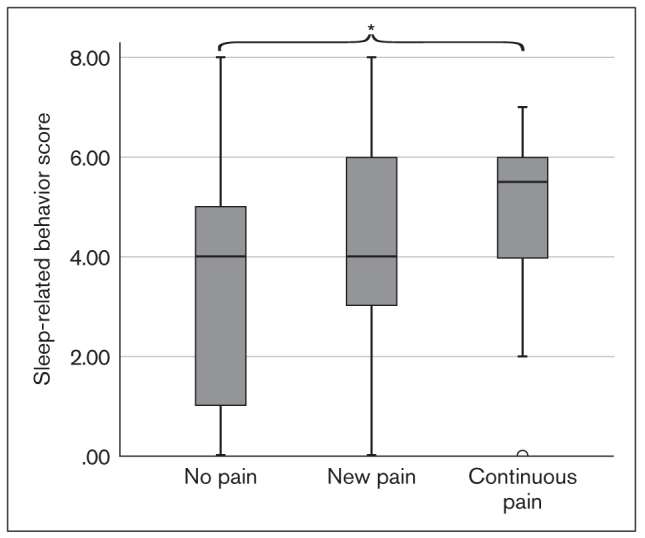
Box plot showing sleep-related oral behavior scores at T1 in the TMD pain groups (*P = .004; independent-samples Kruskal-Wallis test).

**Fig 8:**
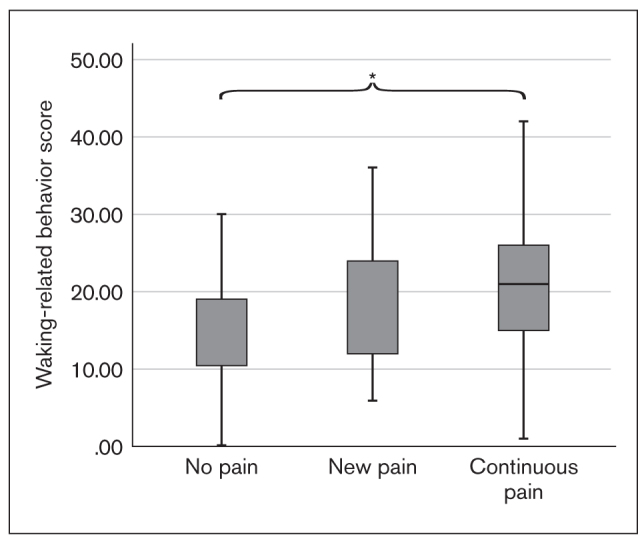
Box plot showing waking-state oral behavior scores at T1 in the TMD pain groups (*P* = .008; independent-samples Kruskal-Wallis test).

Both sleep-related and waking-state oral behavior scores at T1 were notably higher in the continuous pain group than in the no pain group (*P* = .004 and *P* = .008; Figs 7 and 8). In the other pairwise group comparisons (no pain vs new pain, continuous pain vs new pain), a significant difference could not be established.

A considerably higher level of jaw functional limitation was observed at T2 than at T1 (*P* < .001), and, similarly, both sleep-related and waking-state oral parafunction scores were higher at T2 (*P* < .001 for each, Wilcoxon test).

No fundamental difference in terms of changes in anxiety (*P* = .205), depression (*P* = .417), sleep-related (*P* = .378), or waking-state (*P* = .075) parafunctional behaviors between T1 and T2 was found across the three groups (ANOVA). Changes in functional limitation scores were significantly higher in the new pain group than the other groups (*P* = .002).

## Discussion

This study showed that more than 40% of the students who had no pain in the non-exam period reported temporomandibular pain in the subsequent exam period (new pain group), which is a remarkable extent of new pain onset. A corresponding change occurred in the distribution of pain grades, which worsened during the exam period. In the continuous pain group, the pain intensity increased (albeit nonsignificantly) during the exam period, with most such students remaining in Grade 1. Only a small proportion worsened to Grade 3 or 4 with pain-related disability. Simultaneously with changes of pain, the extent of anxiety, depression, and oral parafunctional behaviors increased significantly during the exam period. Functional limitation of the jaw increased significantly in the new pain group, consistent with the known impact of TMD pain on function.

University exams unquestionably act as stressors,^26^ and psychologic distress can affect symptom perception and interpretation, as well as central pain processing.^27,28^ Although the exact mechanisms are not fully understood, an increase in perceived stress levels decreases the pain threshold,^29^ and the exacerbation of pain can be demonstrated in several stress-related disorders, such as primary headaches^30^ and rheumatic diseases.^31^ Anxiety leads to a narrowing of attentional focus and an increased perception of threat and therefore causes people to become more vigilant, scanning both themselves and the environment for potentially threatening cues.^32^ Furthermore, people report more symptoms when they are in a negative mood and are thought to be more vulnerable to illness; they are also more likely to attribute the cause of symptoms to illness.^33^ It is possible that the exacerbation of pain in the present sample is also directly associated with the higher level of psychologic stress related to the exams. The < 10% remission rate in this sample indicates that, in the presence of a predictable (or increasing) stressor load, painful TMDs are a progressive disorder with a potentially lower remission rate than found in a general population such as reported in the OPPERA studies.^34^

In general, university students encounter multiple challenges, such as being away from their families, adult development, and financial constraints.^12^ In the exam periods, the effect of situational stressors can multiply the pressure on students due to the nature of these terms, which is characterized by a constantly heavy workload, lack of time for relaxation,^35^ and a considerable difference in daily routine compared to the non-exam period. Moreover, exam situations often repeat several times over a relatively short interval.

In addition, the extent and quality of coping varies across individuals, which can undoubtedly affect a person’s ability to adapt to a more stressful period.^36^ While over 90% of students in the present study indicated no more than mild levels of anxiety or depression during the non-exam period, that proportion dropped significantly by the exam period. Those who remained in the normal or mild level groups at T2 seemed to have coped much better with exam-related stress than their peers. These findings point out that psychologic states worsened severely in a very high number of students, affecting their daily activities and potentially their exam performance as well. Whether increased anxiety was associated with improved exam performance (eg, via mechanisms of hypervigilance and moderate level of psychologic arousal^37^) or worsened exam performance cannot be determined in the present study. However, as demonstrated by previous studies, factors like self-efficacy and reduced threat appraisal can modulate state anxiety and attenuate the negative effects of stress on exam performance.^38^

The magnitude of associations in the present study suggests that additional but unmeasured variables are important. For example, associations between sleep quality impairment and painful TMDs, especially in those with high Axis II involvement, have been reported by several investigators (eg, Rener-Sitar et al^39^).Disturbed sleep can be present prior to TMD symptoms and is considered to be an indicator of disruptions in the body’s self-regulating mechanisms.^40^ Moreover, findings of previous studies showed that poor sleep quality directly contributes to the development of first-onset TMD and this relationship is mediated by perceived stress.^41^ It is likely that sleep disturbances affected students during the exam period and could have contributed to their reported limitations, influenced their learning abilities as well as their performance in the exams, and directly contributed to the increase in reported pain. Future studies using a DC/TMD Axis II protocol for systematic measurement of potential causal mechanisms regarding new onset or worsening pain should incorporate appropriate measures of sleep dysregulation.

Non-exam–period anxiety levels were significantly higher in the continuous pain group than in the other groups. Similarly, waking-state and sleep-related oral parafunctions were more frequent in the continuous pain group than in the no pain group. It can be hypothesized that students who were inherently anxious were more susceptible to developing persistent pain, and, in their case, non-exam–period anxiety level and oral behaviors were predictors of pain persistence. This finding is in agreement with results of the OPPERA studies, which concluded that a greater extent of oral parafunctions and trait anxiety at enrollment and a change in state anxiety from enrollment to onset period predicts TMD onset.^40^ The same predictive ability for depression scores at enrollment or as a change from T1 to T2 could not be identified; however, the time interval between the two assessments was considerably shorter than the average follow-up time in the OPPERA studies, and this may not have permitted the full expression of depression-like responses to such stressful situations. Although significant increases in all biobehavioral measures and in the proportion of individuals with TMD pain were observed, this study did not find any differences between pain groups regarding changes in the extent of anxiety, depression, or oral parafunctions from T1 to T2.

It seems that pain and the measured biobehavioral variables did not directly influence each other at the time of pain onset, consistent with other findings that highlight the singular importance of pain at the time of pain onset.^42^ However, the relationship between TMDs and biobehavioral variables is considered bidirectional. In the early transition state to pain onset, some of the variables show maladaptive changes across time, which can lead to the onset of TMDs.^40^ At TMD onset, pain is itself the dominant feature, and when it becomes persistent, it will have an effect on psychologic functioning as a stressor. Not surprisingly, these biobehavioral variables have also been recognized as predictors of treatment outcome.^43^ These findings reflect the complexity of TMDs as a chronic pain condition, in particular during the transition phase from acute to chronic. The present study moreover points to the potential types of changes in the very short term as defined within an academic semester. Considerable evidence supports psychologic and behavioral factors in their influence on the processing of pain and therefore cannot be ignored in the diagnostic process and should be appreciated in the management of TMDs.^44^

Based on these results, would students affected by TMD pain require clinical treatment for their possible pain disorder, and should this treatment be done in conjunction with therapy for their anxiety and depressive symptoms? Because case definition was based only on the TMD Pain Screener, the absence of formal diagnosis is an important study limitation. Assuming that clinical examination findings would have been positive for a diagnosis, it is generally agreed that Axis II findings reflect the complexity of the condition,^45^ and for those with high psychosocial involvement, pain management should be aided by a mental health professional.^46–49^ Previous studies have also highlighted the importance of screening university students for anxiety^50^ and providing stress reduction programs.^35^ It is important to note that self-report questionnaires are not adequate for the comprehensive diagnosis of depression and anxiety; rather, formal interview-based evaluation is required. However, these instruments can be extremely useful in identifying individuals with a need for consultation of a mental health care specialist^51,52^ and providing a general measure of psychologic functioning.

A limitation of this study, as just noted, is that the results are based on self-report only and that no clinical examination was performed to confirm the diagnosis of pain-related TMDs. The COVID-19 pandemic imposed restrictions in Hungarian educational settings, which severely limited personal contact with students, especially in clinical settings. However, the Pain Screener instrument used is a reliable screening tool in providing data about painful TMDs, such that the diagnosis of arthralgia and/or myalgia can be established when the location of pain is in the masticatory structures and is modified by jaw movement, function, or parafunction.^53^ Because the excellent reliability of the TMD Pain Screener was tested on chronic TMD cases, it was expected that the performance of the instrument may be different in the sample with new onset painful TMDs. Based on our experience in using the instrument in clinical settings, this may have resulted in an underestimation regarding the number of new cases in the exam period, as some students may have already resolved by the time of answering the questionnaire for the second occasion. It is also possible that, in such individuals, the effect of pain on function was minimal, and their response to the related questions of the Pain Screener was “no,” which precluded them from being classified as a new pain case. Another limitation is the possible order of effects imposed by measuring everyone first during non-exam and then during exam periods or, equivalently, the absence of a control group measured during two non-exam periods, which limits the causal conclusions. Additionally, a stress reaction was inferred as based on how exam periods are typically regarded, but a direct measure of stressfulness with coping response, such as using the Perceived Stress Scale,^54^ would provide greater insight into how pain, anxiety, depression, and oral behaviors interact during such periods. Similarly, no instruments were used to measure changes in quality of life or exam performance. A final limitation is that the observed dropout from initial enrollment to final sample is a possible source of bias in the outcomes. Refusing to participate or losing interest for the second time answering may have been the reasons why several students were lost from the initial sample, which is the nature of most observational studies. The requirement that all items had to be answered for the questionnaire to be submitted may have also contributed to dropout. Any such dropout would be important only if it was associated with the variables of interest.

The primary strength of this study is that it approaches a complex health problem (ie, temporomandibular pain and stress-related psychologic disorders) in a comprehensive and ecologic manner. Axis II of the DC/TMD was developed to complement physical diagnosis with the biopsychosocial aspects of the disorder, as these factors can impact management of TMD patients.^55^ The present findings contribute congruent findings to the existing literature regarding stressfulness of professional education,^15,56,57^ highlighting the general importance of improving university students’ mental well-being. The present findings also highlight that even acute onset of painful TMDs warrants consideration for using a multidisciplinary approach to management of both pain and stress. Moreover, future research is required to clarify how the presence of orofacial pain and simultaneous Axis II involvement in students affect their academic performance, including exam results and professional development.

## Conclusions/Key Findings

The results of this study suggest the following:

- The proportion of individuals with orofacial pain significantly increased from the non-exam period to the exam period.
- Higher levels of anxiety and oral behaviors in the non-exam period were predictors for persisting TMD pain.
- Similarly, levels of biobehavioral variables were significantly higher in the exam period.
